# Correlation between Skip N2 Metastases and SUV_max_, Long Diameter of Tumor, and Ki67 Expression in Patients with Non-Small-Cell Lung Cancer

**DOI:** 10.1155/2020/9298358

**Published:** 2020-04-25

**Authors:** Wang Jian, Peng Ming-ya, Xu Long-bao, Zhao Jun, Shao Guo-qiang

**Affiliations:** ^1^Oncology Center, Jiangyin People's Hospital, Affiliated Hospital of Southeast University, Jiangyin 214400, China; ^2^Department of Nuclear Medicine, Changzhou Second People's Hospital Affiliated to Nanjing Medical University, Changzhou 213003, China; ^3^Department of Nuclear Medicine, Nanjing First Hospital, Nanjing Hospital Affiliated to Nanjing Medical University, Nanjing 210006, China

## Abstract

**Background:**

We aim at investigating the correlation between skip N2 metastases (SN2) and SUV_max_, long diameter of tumor mass after ^18^F-FDG PET/CT, and pathological Ki67 expression in patients with non-small-cell lung cancer (NSCLC).

**Methods and Results:**

We retrospectively analyzed the factors that might affect the pathogenesis of SN2 in these patients. The clinical SN2 symptoms in patients with squamous carcinoma or adenocarcinoma were investigated. The work curve was utilized to analyze the optimal cutoff value for the SUV_max_ and long diameter of tumor. Multivariate analysis revealed that high expression of Ki67 was a risk factor for mediastinal SN2 (OR = 1.042, 95% CI: 1.009-1.076). Subgroup analysis indicated that the SUV_max_ of the non-SN2 group was significantly higher than that of the SN2 group in patients with squamous carcinoma (16.3 ± 6.0 vs. 10.7 ± 5.6, *P* = 0.026). In the patients with adenocarcinoma, the long diameter of tumor in the SN2 group was significantly longer than that of the non-SN2 group (43.8 ± 16.3 mm vs. 30.1 ± 13.8 mm, *P* = 0.032). The Ki67 expression in the SN2 group was significantly higher than that of the non-SN2 group (51.7 ± 24.0 vs. 30.0 ± 19.2, *P* = 0.028).

**Conclusions:**

The differences of clinical features of the patients in the SN2 group and non-SN2 group in the NSCLC patients were associated with the pathological subtypes, which were featured by lower SUV_max_ in the SN2 of the squamous carcinoma, and longer diameter of SN2 in the adenocarcinoma patients.

## 1. Introduction

Lung cancer is the leading cause of cancer-related death worldwide, leading to severe threats to the public health [1]. Non-small-cell lung cancer (NSCLC) is the predominant pathological type of lung cancer accounting for about 85% in total [2]. For the NSCLC patients, mediastinal or pulmonary lymph node metastasis is a crucial factor for the establishment of treatment regimen and judgment of outcome. The conventional lymph node metastasis refers to the metastasis of cancer cells from the peripheral lymph nodes to the mediastinal lymph nodes through the hilum of lung, while partial patients showed skip N2 metastases (SN2) in the mediastinal lymph nodes rather than the hilar lymph nodes [3]. In the eighth version of the TNM staging guidelines for lung cancer, SN2 is classified into the N2a1 substage [4, 5]. To date, rare studies have been focused on the evaluation of SN2 using the ^18^F-FDG PET/CT scan. The uptake of ^18^F-FDG in the primary lesions of NSCLC is considered as an independent risk factor for lymph node metastasis [6, 7]. In addition, the tumor volume and pathological Ki67 expression were closely related to the lymph node metastasis in the NSCLC patients [8, 9]. In this study, we retrospectively analyzed 65 NSCLC cases confirmed with pulmonary, ipsolaterally hilar, or mediastinal lymph nodes, with an aim at investigating the correlation between SN2 and the maximum standardized uptake value (SUV_max_) by ^18^F-FDG PET/CT, Ki67 expression on SN2, and the clinical features of SN2 in patients with different pathological types (i.e., squamous carcinoma or adenocarcinoma).

## 2. Materials and Methods

### 2.1. Clinical Data

**Figure 1:**
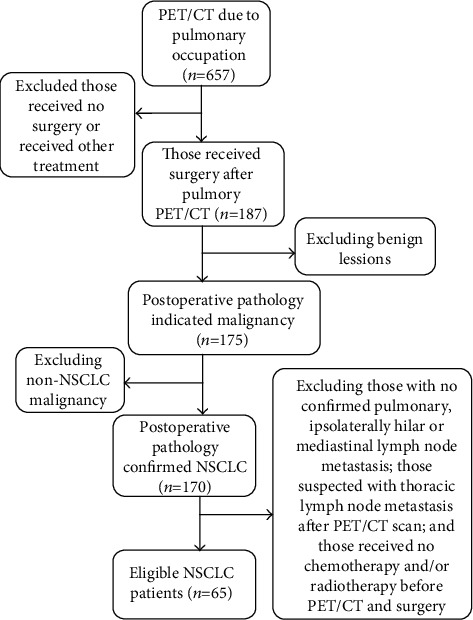
Study flowchart.

A total of 65 NSCLC patients, who received ^18^F-FDG PET/CT within 2 weeks before surgery in our hospital between January 2016 and December 2018, were included in this study. The inclusion criteria were as follows: those aged ≤ 75 yrs; those confirmed with pulmonary, ipsolaterally hilar, or mediastinal lymph node metastasis; suspected with thoracic lymph node metastasis after PET/CT scan; received no chemotherapy and/or radiotherapy before PET/CT and surgery; and those with no other malignancies. Those aged > 75 yrs, with surgical contraindications or mediastinal/supraclavicular lymph node metastasis (N3) and distal metastasis (M_1_), were excluded from the study (Figure 1). Each patient signed the informed consent. The study protocols were approved by the Ethical Committee of Changzhou Second People's Hospital Affiliated to Nanjing Medical University.

### 2.2. ^18^F-FDG PET/CT

PET/CT was performed using the uMI780 112 facility (United Imaging, Shanghai, China). Prior to the scan, all the patients were required in a fasting condition for 6 hrs and then intravenous injection of ^18^F-FDG (0.12 mCi/kg) was given. After a 60 min rest, whole-body PET/CT was carried out. The reconstructed images were collected to obtain the SUV_max_. Two experienced radiologists reviewed the images in a blinded manner. The SUV_max_ of the primary lesions was determined using the multiple ellipse region of interest (ROI) from cross-section, which meant the semiquantitative analysis for the maximum value. A SUV_max_ of ≥2.5 was defined as abnormality [10]. In addition, the clinical files were taken into consideration for the diagnosis.

### 2.3. Ki67 Expression Determination

The immunohistochemistry findings of Ki67 were obtained from the postoperative pathology. Positive staining was defined as the presence of brown granules in the nucleus.

### 2.4. Statistical Analysis

The MedCalc software package was utilized for the statistical analysis. The pathological findings were used as the gold standards. On this basis, the sensitivity and specificity of SN2 in NSCLC patients were compared and calculated using PET/CT. The Kolmogorov-Smirnov was used to evaluate the normal distribution of the data. The measurement data that were normally distributed were presented as mean ± standard deviation. Intergroup comparison was given using the independent *t*-test, Satterthwaite *t*-test, or Mann–Whitney nonparameter test. Chi-squared test or Fisher's exact test was utilized for the comparison of the intergroup sample rate. The univariate and multivariate logistic regression analysis was used for the evaluation of the risk factors of SN2. The receiver operating characteristic (ROC) curvature was used for the analysis of the best cutoff value for the continuous data. *P* < 0.05 was considered statistically significant.

## 3. Results

### 3.1. Clinical Data

**Figure 2:**
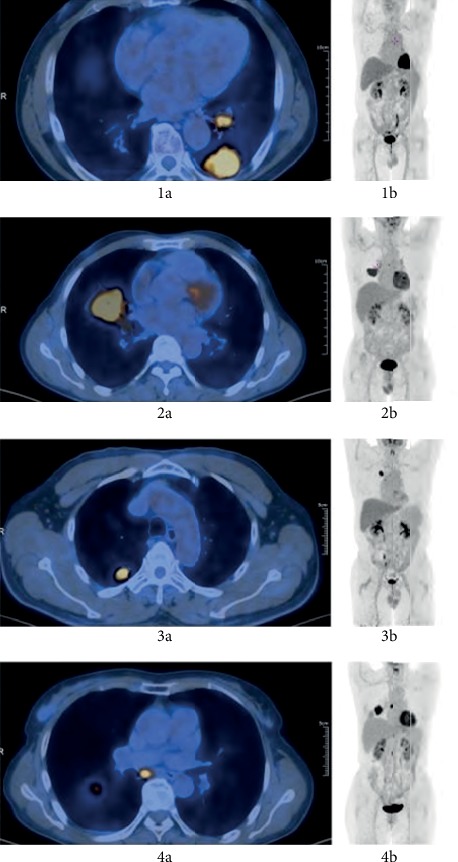
The fusion image (a) and MIP image (b) of patients with pulmonary adenocarcinoma and squamous carcinoma. 1a and 1b: a 66-year-old male patient presented with squamous carcinoma in the inferior lobe of the left lung combined with hilar lymph node metastasis. The SUV_max_ was about 15.1 and Ki67 was about 70, which was presented as non-SN2. 2a and 2b: a 55-year-old male patient showed squamous carcinoma in the middle lobe of the right lung combined with paratracheal lymph nodes. No lymph node metastasis was observed in the right hilum of the lung. The SUV_max_ was about 10.8, and Ki67 was about 60, which was presented as SN2. 3a and 3b: a 67-year-old male patient presented to our hospital due to adenocarcinoma in the superior lobe of the right lung combined with right hilar lymph node metastasis. The long diameter of tumor was about 24.0 mm, and Ki67 was about 30, which was presented as non-SN2. 4a and 4b: a 64-year-old female patient showed adenocarcinoma in the inferior lobe of the right lung combined with lymph node metastasis beneath the eminence. There were no lymph node metastases in the right hilum of the lung. The long diameter of the tumor was about 47.0 mm, and Ki67 was about 70, which was presented as SN2.

Using the pathological data as the golden standard for the diagnosis of lymph node metastasis, 65 NSCLC patients were included in this study (Table 1). The pathological types consisted of squamous carcinoma (*n* = 26), adenocarcinoma (*n* = 36), adenosquamous carcinoma (*n* = 2), and lymphoepithelioma (*n* = 1). The number of patients with non-SN2 (e.g., hilar lymph node metastasis and/or mediastinal lymph node metastasis) and SN2 was 46 and 19, respectively. In the squamous carcinoma patients, 16 (61.5%) showed non-SN2 and 10 (38.5%) showed SN2 (Figure 2, 2a and 2b). In the adenocarcinoma patients, 28 (77.8%) showed non-SN2 and 8 showed SN2 (Figure 2, 2c and 2d). For the patients with other pathological types (*n* = 3), 2 (66.7%) showed non-SN2 and 1 (33.3%) showed SN2, respectively.

### 3.2. Clinical Analysis of SN2 in the Whole Group

In the patients with SN2, the expression of Ki67 was higher than that of the non-SN2 patients (*P* = 0.008). No statistical differences were noticed in the age, gender, primary lesion site, history of smoking, pleural involvement, pathological type, and long diameter (*P* > 0.05, Table 2). Multivariate analysis showed that Ki67 elevation was an important factor for the pathogenesis of SN2 (OR = 1.042, 95% CI: 1.009-1.076).

### 3.3. Comparison of Clinical Features between SN2 and Non-SN2 Groups in Squamous Carcinoma Patients

The SUV_max_ in the squamous carcinoma patients with SN2 and the non-SN2 group was 10.7 ± 5.6 and 16.3 ± 6.0, respectively. The SUV_max_ in the non-SN2 patients with squamous carcinoma was significantly higher than that of the SN2 cases (16.3 ± 6.0 vs. 10.7 ± 5.6, *t* = 2.369, *P* = 0.026). The long diameter of the squamous carcinoma patients with SN2 and the non-SN2 group was 48.1 ± 25.5 mm and 45.8 ± 27.5 mm, respectively. The long diameter of tumor in the SN2 patients and non-SN2 patients showed no statistical differences (48.1 ± 25.5 mm vs. 45.8 ± 27.5 mm, *t* = 0.771, *P* = 0.578). The Ki67 in the squamous carcinoma patients with SN2 and the non-SN2 group was 65.0 ± 5.4 and 61.7 ± 18.0, respectively. Meanwhile, no statistical differences were noticed in the Ki67 expression in SN2 patients and non-SN2 patients (65.0 ± 5.4 vs. 61.7 ± 18.0, *t* = 0.505, *P* = 0.619, Table 3).

### 3.4. Comparison of Clinical Features between SN2 and Non-SN2 Groups in Adenocarcinoma Patients

The SUV_max_ in the adenocarcinoma patients with SN2 and the non-SN2 group was 11.8 ± 3.6 and 10.9 ± 5.6, respectively. The SUV_max_ in the SN2 patients with adenocarcinoma showed no differences compared with that of the non-SN2 cases (11.8 ± 3.6 vs. 10.9 ± 5.6, *t* = 0.411, *P* = 0.684). The long diameter in the adenocarcinoma patients with SN2 and the non-SN2 group was 43.8 ± 16.3 mm and 30.1 ± 13.8 mm, respectively. The long diameter of tumor in the SN2 patients was significantly higher than that of non-SN2 patients (43.8 ± 16.3 mm vs. 30.1 ± 13.8 mm, *t* = 2.244, *P* = 0.032). The Ki67 in the adenocarcinoma patients with SN2 and the non-SN2 group was 51.7 ± 24.0 and 30.0 ± 19.2, respectively. Meanwhile, no statistical differences were noticed in the Ki67 expression in SN2 patients and non-SN2 patients (51.7 ± 24.0 vs. 30.9 ± 19.2, *t* = 2.332, *P* = 0.028, Table 4).

### 3.5. Efficiency of ^18^F-FDG PET/CT on Evaluation of SN2

**Figure 3:**
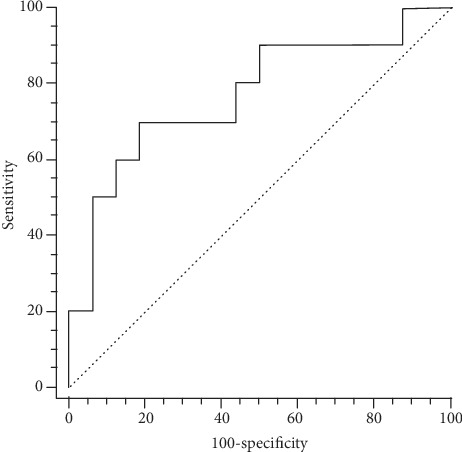
The ROC curvature of the SN2 based on the SUV_max_ in patients with squamous carcinoma.

**Figure 4:**
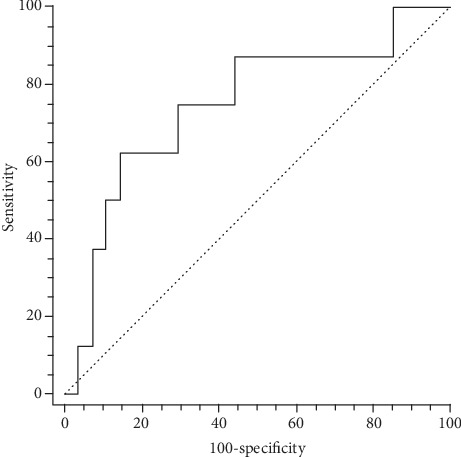
ROC curvature of the SN2 based on the long diameter of tumor in patients with adenocarcinoma.

For the patients with squamous carcinoma, the sensitivity, specificity, and accuracy for SN2 using PET/CT was 80.0% (8/10), 93.8% (15/16), and 88.5% (23/26), compared to the gold standard (i.e., pathological report). The ROC curvature showed that the maximal AUC (AUC = 0.769, *P* = 0.01) was obtained when the SUV_max_ was 11.4 (Figure 3). The generated sensitivity and specificity was 70.0% and 81.3%, respectively. Using the combination of PET/CT and cutoff value of SUV_max_ (<11.4) for the diagnosis of SN2 (*n* = 6) and non-SN2 (*n* = 26), the sensitivity and specificity for SN2 of the squamous cancer was 60.0% (6/10) and 100% (16/16), respectively. For the patients with adenocarcinoma, the sensitivity, specificity, and accuracy for PET/CT-based SN2 was 62.5% (5/8), 92.9% (26/28), and 86.1% (31/36), compared to the gold standard. The maximal AUC (AUC = 0.745, *P* = 0.025) was obtained in the presence of a long tumor diameter of 41.6 mm (Figure 4). The sensitivity and specificity was 62.5% and 85.2%, respectively. Based on the combination of PET/CT and the cutoff value of long tumor diameter (>41.6 mm), the sensitivity and specificity for SN2 of the adenocarcinoma patients were 50.0% (4/8) and 100% (28/28), respectively.

## 4. Discussion

The mediastinal lymph node metastasis in NSCLC patients is usually through multiple classic pathways, which involves the dissemination of primary cancer cells to the pleura and hilar lymph nodes (N1), the ipsolateral mediastinal lymph nodes (N2), and finally the contralateral mediastinal lymph nodes and supraclavicular lymph nodes (N3). In a previous study, Riquet et al. [3] reported that there was direct lymphatic vasculature to the diaphragmatic lymph nodes in the inferior lung segment near the pleura. In the cases of lymphatic metastasis, it may surpass the pulmonary lymph nodes and hilar lymph nodes, which was defined as SN2. Such phenomenon was reported to show an incidence of about 20%-40% [3, 11–13]. Meanwhile, Zhao et al. [13] revealed that SN2 commonly existed regardless of the surgical options or clearance of lymph nodes. Nowadays, there are still some disputes on the evaluation of SN2 in the NSCLC patients, including the incidence [14], metastatic mechanism [11], and prognosis [13]. In the past decades, the SN2 was mainly evaluated based on the NSCLC views. If possible, accurate evaluation should be given in the pathological subtypes, which may be helpful to illustrate the clinical symptoms differences.

^18^F-FDG PET/CT is considered as the gold standard for the noninvasive imaging evaluation for the clinical staging of NSCLC [15]. Such technique could present the morphological parameters of lymph nodes and judge the malignant or benign types of the glucose metabolism in lymph nodes. The corresponding semiquantitative index was illustrated as SUV_max_. According to the previous study, SUV_max_ was correlated to the lymphatic invasion and lymph node metastasis [6]. To date, rare studies have been focused on the relationship between SUV_max_ and SN2. In this study, there were no statistical differences in the SUV_max_ and long tumor diameter in the SN2 group and non-SN2 group. However, for the patients with squamous carcinoma, pathological subtype analysis showed that the SUV_max_ in the non-SN2 group was superior to that of the SN2 group (*P* = 0.026). In the adenocarcinoma patients, the long diameter of tumor in the SN2 group was significantly longer than that of the non-SN2 group (*P* = 0.032). Therefore, SN2 was associated with SUV_max_ in the patients with squamous carcinoma and was associated with long diameter in the adenocarcinoma patients. The differences of clinical features between the squamous carcinoma and adenocarcinoma may be related to the biological behaviors of various pathological tumors.

Most of the squamous carcinoma was in a central type. Its conventional metastasis pathway was mainly featured by the lymphatic canal between the lesions and the mediastinum. Those with a higher SUV_max_ presented a high cancer proliferation and metastasis, together with elevation in the peripheral angiogenesis and generation of lymphatic vessels [16, 17], which then promoted the conventional metastasis velocity that was even a faster entry to the mediastinum than the SN2. Therefore, the possibility of SN2 detection may be reduced. The adenocarcinoma were mainly in a peripheral type, with the hematogenous metastasis as the main type. A larger tumor volume presented a close distance between the lesions to the peripheral pleura, and the peripheral vessels and lymphatic capillary between the lobes were more abundant. This contributed to the increased possibility of cancer cells into the SN2 metastasis pathway (subpleural lymphatic vessels), which was featured by a correlation between adenocarcinoma SN2 and long diameter of tumor.

Ki67, a cell cycle related protein, has been listed as an effective index for evaluating the proliferation of cancer cells and the treatment prognosis [18, 19]. In a previous study, Ki67 expression was reported to be closely related to the lymph node metastasis and tumor staging, together with the prognosis of adenocarcinoma patients [9]. To our best knowledge, there are no studies focused on the relationship between Ki67 expression and SN2. In this study, patients with high expression of Ki67 were likely to present SN2. The potential causes may be related to the fact that the entry of cancer cells into the lymphatic vasculature was regulated by the proliferation of cancer cells, as well as the adhesion of cancer cells and the lymphatic epithelial cells [20, 21]. The cancer cells with high expression of Ki67 showed higher proliferation capacity, which showed a higher potency of rapid entry to the lymphatic vasculature that was featured by high possibility of SN2.

SN2 has been commonly acknowledged to be associated with satisfactory prognosis; however, some studies proposed no association between SN2 and the prognosis [11, 14]. The disputes are mainly stemmed from studies involving only single-station SN2 [15]. These studies were mainly focused on the NSCLC, other than the squamous carcinoma or adenocarcinoma. Nowadays, it has been reported that NSCLC patients with single-station SN2 showed similar overall survival, relapse-free survival, and N1 staging, and surgery is considered to be appropriate for the treatment [18]. Therefore, clinical evaluation of SN2 is of prime importance for the diagnosis, treatment, and prognosis of certain disease. In this study, there were false positivity and negativity when evaluating the SN2 in those with squamous carcinoma or adenocarcinoma using the PET/CT technique. The diagnostic specificity for the technique combined with threshold of SUV_max_ or combined with the longest tumor diameter was 100%, respectively. Besides the detection of SN2, it would contribute to the diagnosis of single-station or multiple-station SN2, which may provide benefits to the preoperative SN2.

There are really some limitations in this study. The conclusions in this study are required to be confirmed by multicentered, large sample studies. In addition, only preliminary investigation was given to the PET/CT findings of the SN2. In the future, studies are needed to fully illustrate the pathogenesis of SN2 and its clinical significance.

In summary, SN2 is common among NSCLC patients. It is of prime importance in the improvement of the TNM staging, treatment regimen preparation, and judgment of treatment prognosis. In this study, the differences of clinical features of the patients in the SN2 group and non-SN2 group in the NSCLC patients were associated with the pathological subtypes, which were featured by lower SUV_max_ in the SN2 of the squamous carcinoma, and longer long diameter of SN2 in the adenocarcinoma patients. PET/CT provided additional information of preoperative SN2 for the diagnosis of NSCLC.

## Figures and Tables

**Table 1:** General characteristics of the 65 NSCLC patients.

Variables	*N* (%)
Age	
<60 yrs	16 (24.6%)
≥60 yrs	49 (75.4%)
Pathological type	
Adenocarcinoma	36 (55.4%)
Squamous carcinoma	26 (40.0%)
Adenosquamous carcinoma	2 (3.1%)
Lymphoma-like carcinoma	1 (1.5%)
T staging	
T_1_	22 (33.8%)
T_2_	28 (40.3%)
T_3_	12 (19.4%)
T_4_	3 (6.5%)
N staging	
N1a	14 (21.5%)
N1b	4 (6.2%)
N2a1	19 (29.2%)
N2a2	15 (23.1%)
N2b	13 (20.0%)
TNM staging	
IIB	22 (33.8%)
IIIA	34 (52.3%)
IIIB	9 (13.8%)

NSCLC: non-small-cell lung cancer.

**Table 2:** Clinical symptoms of patients in SN2 and non-SN2 groups.

Variables	SN2 group (n = 19)	Non-SN2 group (*n* = 46)	*t*/*χ*^2^	*P* value
Age	63.1 ± 6.2	61.9 ± 9.0	0.485	0.629
Gender			—	0.14
Male	16	29		
Female	3	17		
Smoking			—	0.803
Yes	4	11		
No	15	35		
Tumor site			—	0.658
Right upper lobe	6	12		
Middle lobe	2	5		
Right low lobe	3	12		
Left upper lobe	4	7		
Left low lobe	4	10		
Pleural invasion			—	0.875
Yes	3	8		
No	16	38		
Pathological type			1.785	0.182
Squamous carcinoma	10	16		
Nonsquamous carcinoma	9	30		
Ki67 expression	60.5 ± 16.5	41.2 ± 20.1	2.828	0.008
Long diameter of tumor (mm)	44.8 ± 20.2	35.2 ± 19.8	1.765	0.123
Tumor SUV_max_	11.2 ± 5.0	12.6 ± 6.4	0.823	0.396

SUV_max_: maximum standardized uptake value. Fisher's exact method.

**Table 3:** Comparison of clinical features in the squamous carcinoma patients with SN2 and the non-SN2 group.

	SN2 group (*n* = 10)	Non-SN2 group (*n* = 16)	*t*	*P* value
SUV_max_, squamous carcinoma	10.7 ± 5.6	16.3 ± 6.0	2.369	0.026
Long diameter (mm), squamous carcinoma	48.1 ± 25.5	45.8 ± 27.5	0.771	0.578
Ki67 expression	65.0 ± 5.4	61.7 ± 18.0	0.505	0.619

**Table 4:** Comparison of clinical features of adenocarcinoma patients with SN2 and the non-SN2 group.

	SN2 group (*n* = 8)	Non-SN2 group (*n* = 28)	*t*	*P* value
SUV_max_, adenocarcinoma	11.8 ± 3.6	10.9 ± 5.6	0.411	0.684
Long diameter (mm), adenocarcinoma	43.8 ± 16.3	30.1 ± 13.8	2.244	0.032
Ki67 expression	51.7 ± 24.0	30.0 ± 19.2	2.332	0.028

## Data Availability

All the data were available upon appropriate request.

## References

[1] Bray F., Ferlay J., Soerjomataram I., Siegel R. L., Torre L. A., Jemal A. (2018). Global cancer statistics 2018: GLOBOCAN estimates of incidence and mortality worldwide for 36 cancers in 185 countries. *CA: a Cancer Journal for Clinicians*.

[2] Hochhegger B., Alves G. R., Irion K. L. (2015). PET/CT imaging in lung cancer: indications and findings. *Jornal Brasileiro de Pneumologia*.

[3] Riquet M., Assouad J., Bagan P. (2005). Skip mediastinal lymph node metastasis and lung cancer: a particular N2 subgroup with a better prognosis. *The Annals of Thoracic Surgery*.

[4] Hattori A., Takamochi K., Oh S., Suzuki K. (2019). New revisions and current issues in the eighth edition of the TNM classification for non-small cell lung cancer. *Japanese Journal of Clinical Oncology*.

[5] Chansky K., Detterbeck F. C., Nicholson A. G. (2017). The IASLC lung cancer staging project: external validation of the revision of the TNM stage groupings in the eighth edition of the TNM classification of lung cancer. *Journal of Thoracic Oncology*.

[6] Higashi K., Ito K., Hiramatsu Y. (2005). 18F-FDG uptake by primary tumor as a predictor of intratumoral lymphatic vessel invasion and lymph node involvement in non-small cell lung cancer: analysis of a multicenter study. *Journal of Nuclear Medicine*.

[7] Miyasaka Y., Suzuki K., Takamochi K., Matsunaga T., Oh S. (2013). The maximum standardized uptake value of fluorodeoxyglucose positron emission tomography of the primary tumour is a good predictor of pathological nodal involvement in clinical N0 non-small-cell lung cancer. *European Journal of Cardio-Thoracic Surgery*.

[8] Yankelevitz D., Wisnivesky J. P., Henschke C. I. (2005). Stage of lung cancer in relation to its size: part 1. Insights. *Chest*.

[9] del Gobbo A., Pellegrinelli A., Gaudioso G. (2016). Analysis of NSCLC tumour heterogeneity, proliferative and 18F-FDG PET indices reveals Ki67 prognostic role in adenocarcinomas. *Histopathology*.

[10] Schmidt-Hansen M., Baldwin D. R., Hasler E. (2014). PET-CT for assessing mediastinal lymph node involvement in patients with suspected resectable non-small cell lung cancer. *Cochrane Database of Systematic Reviews*.

[11] Gorai A., Sakao Y., Kuroda H. (2015). The clinicopathological features associated with skip N2 metastases in patients with clinical stage IA non-small-cell lung cancer. *European Journal of Cardio-Thoracic Surgery*.

[12] Wang L., Zhan C., Gu J. (2019). Role of skip mediastinal lymph node metastasis for patients with resectable non–small-cell lung cancer: a propensity score matching analysis. *Clinical Lung Cancer*.

[13] Zhao J., Li J., Li N., Gao S. (2018). Clinical significance of skipping mediastinal lymph node metastasis in N2 non-small cell lung cancer. *Journal of Thoracic Disease*.

[14] Misthos P., Sepsas E., Athanassiadi K., Kakaris S., Skottis I. (2004). Skip metastases: analysis of their clinical significance and prognosis in the IIIA stage of non-small cell lung cancer. *European Journal of Cardio-Thoracic Surgery*.

[15] de Castro A. B. G., Domínguez J. F., Bolton R. D. (2017). PET-CT in presurgical lymph node staging in non-small cell lung cancer: The importance of false-negative and false-positive findings. *Radiología*.

[16] Surov A., Meyer H. J., Wienke A. (2018). Standardized uptake values derived from 18F-FDG PET may predict lung cancer microvessel density and expression of KI 67, VEGF, and HIF-1*α* but not expression of cyclin D1, PCNA, EGFR, PD L1, and p53. *Contrast Media & Molecular Imaging*.

[17] Kocael A., Vatankulu B., Şimşek O. (2016). Comparison of (18)F-fluorodeoxyglucose PET/CT findings with vascular endothelial growth factors and receptors in colorectal cancer. *Tumour Biology*.

[18] Wen S., Zhou W., Li C. M. (2015). Ki-67 as a prognostic marker in early-stage non-small cell lung cancer in Asian patients: a meta-analysis of published studies involving 32 studies. *BMC Cancer*.

[19] Ács B., Zámbó V., Vízkeleti L. (2017). Ki-67 as a controversial predictive and prognostic marker in breast cancer patients treated with neoadjuvant chemotherapy. *Diagnostic Pathology*.

[20] Fidler I. J. (1989). Origin and biology of cancer metastasis. *Cytometry*.

[21] Sleeman J. P., Thiele W. (2009). Tumor metastasis and the lymphatic vasculature. *International Journal of Cancer*.

